# *In vitro* removal of paraquat and diquat from aqueous media using raw and calcined basil seed

**DOI:** 10.1016/j.heliyon.2021.e07644

**Published:** 2021-07-27

**Authors:** Yugo Uematsu, Fumihiko Ogata, Noriaki Nagai, Chalermpong Saenjum, Takehiro Nakamura, Naohito Kawasaki

**Affiliations:** a Faculty of Pharmacy, Kindai University, 3-4-1 Kowakae, Higashi-Osaka, Osaka 577-8502, Japan; b Faculty of Pharmacy, Chiang Mai University, Suthep Road, Muang District, Chiang Mai, 50200, Thailand; c Cluster of Excellence on Biodiversity-based Economics and Society (B.BES-CMU), Chiang Mai University, Suthep Road, Muang District, Chiang Mai, 50200, Thailand; d Antiaging Center, Kindai University, 3-4-1 Kowakae, Higashi-Osaka, Osaka 577-8502, Japan

**Keywords:** Basil seed, Paraquat, Diquat, Adsorption

## Abstract

Raw and calcined basil seeds (BS and BS1000, respectively) were evaluated for their ability to remove herbicides such as paraquat and diquat. The physicochemical properties of BS and BS1000 were determined and the effects of contact time and initial concentration on paraquat and diquat adsorption were assessed. After calcination treatment, the number of pores in BS increased, and the specific surface area was increased from 0.265 to 86.902 m^2^ g^−1^. The quantity of herbicides adsorbed using BS1000 was greater than that using either BS or medicinal-grade carbon. Additionally, the adsorption quantity increased with the increase in contact time and initial concentration of herbicide. Therefore, BS1000 is a potential resource for the removal of herbicides. Moreover, BS and BS1000 exhibited the capacity for herbicide adsorption in simulated intestinal fluid.

## Highlights

• Calcined basil seed (BS1000) showed the adsorption capability of herbicides.
• Binding energy of carbon onto BS1000 surface increased after adsorption.
• Herbicides were removed using BS1000 from simulated intestinal fluid.

## 1 Introduction

The Sustainable Development Goals (SDGs) were initiated and approved by all member states of the United Nations in 2015 to achieve the goals of establishing a sustainable society and prosperity for all. In particular, Goal 7 (Affordable and clean energy) and Goal 12 (Responsible consumption and production) aim to develop useful recycling technologies for reducing wastes globally (The United Nations, 2021). Increasing attention is currently being focused on waste biomass such as agricultural waste as resource material with various applications. Although waste biomass is generated worldwide, the entire amount may not be usefully recycled and safely disposed annually. Therefore, the development of recycling technology for waste biomass is urgently needed. Previous studies have reported that waste biomass such as mangosteen, wheat bran, basil seed, and agriculture wastes showed the capacity to adsorb harmful substances such as mercury, chromium, strontium, cesium, molybdenum, and dyes from aqueous media (Ogata et al., 2018, 2020a, 2020b; Sivarama Krishna et al., 2014; Somasekhara Reddy et al., 2012; Uematsu et al., 2020). These results indicate that the conversion from waste biomass to an adsorbent is one of the most useful techniques for reducing these wastes.

The use of pesticides in agricultural fields is very important as they are a useful means of reducing production efforts and increasing agricultural productivity (Junthip et al., 2019). Paraquat and diquat are widely used and classified as non-selective contact viologen herbicides that kill plant tissue on contact (Kumari et al., 2019). Moreover, pesticides such as paraquat and diquat are sometimes consumed to commit suicide owing to their characteristics (Seok et al., 2009). Studies previously have reported that paraquat is connected to the development of Parkinson's disease, lung fibrosis, and damage to the kidney, liver, and esophagus (Duarte et al., 2008; Gawarammana and Buckley, 2011; Vaccari et al., 2017), whereas diquat can cause severe irritation of the stomach, throat, mouth, and eye (Jones and Vale, 2000). Therefore, each pesticide was classified as a moderate toxic compound (Class II) by the World Health Organization (WHO). The lethal dose (LD_50_) values of paraquat and diquat were reported in the concentration of 150 mg kg^−1^ and 231 mg kg^−1^, respectively (Mehmandost et al., 2020; World Health Organization, 2010). Moreover, a previous study reported the relationship between the plasma paraquat concentration and survival prognosis (Senarathna et al., 2009). Therefore, the removal of paraquat and diquat residues from the environment is very important for preventing human diseases.

Till date, various physicochemical approved procedures such as reverse osmosis (Sir et al., 2015), electrocoagulation (Beninca et al., 2016), chemical oxidation (Fan et al., 2017), and adsorption (Ahmaruzzaman and Gupta, 2011) have been demonstrated as the potential techniques for the removal of herbicides, including paraquat and diquat. Among them, adsorption is the most common treatment owing to cost-effectiveness, low energy consumption, and simple operation (Duman et al., 2019). However, there are few reports regarding the *in vitro* assessment of the removal of herbicides such as paraquat and diquat using adsorbents prepared from waste biomass.

Therefore, in this study, waste biomass namely basil seeds was focused for the removal of paraquat and diquat. *Ocimum basilicum* L. (basil) is a common and well-known herb (Lodhi et al., 2019). *Ocimum* comprises approximately 150 species of herbs and shrubs (Khazaei et al., 2014). The mucilage of *O. basilicum* composed of various carbohydrates such as D-glucose, D-galactose, D-mannose, L-rhamnose, glucomannan, pectins, and hemicellulose. Additionally, it includes small amounts of non-polysaccharides such as protein, minerals, and fats (Ahmas et al., 2015). In particular, when the outer pericarp of basil seed is soaked in an aqueous solution, it swells into a gelatinous mass (Azuma and Sakamoto, 2003; Naji-Tabasi and Razavi, 2017). The outer of the swollen seeds composed of pectinous matrix which consisting of considerable amounts of unesterified galacturonic acid with a large capacity of water adsorption (Abraham and Werker, 1972). Basil has been traditional used as a medicinal plant to treat ailments such as kidney malfunction (Simon et al., 1999). A previous study reported that basil seed had the capacity to adsorb strontium and cesium from aqueous media (Uematsu et al., 2020) indicating that the potential of basil seed as a waste-biomass material (adsorbent) for the removal of harmful chemicals. In addition, this report elucidated that the adsorption capacity of calcined basil seed was greater than that of virgin basil seed for harmful cation chemicals from aqueous phase. Because the specific surface area and the number of surface functional groups, which directly affect the adsorption capacity, of calcined basil seed were higher than those of virgin basil seed. Finally, basil seed is edible and therefore safe for human health. However, there are no reports on the evaluation of the relationship between virgin or calcined basil seed and herbicides such as paraquat and diquat in an *in vitro* system. Therefore, if herbicides could be removed using basil seed in an *in vitro* system, its value and feasibility would increase substantially and useful information could be obtained to elucidate a mechanism for the adsorption of herbicides to prevent human diseases.

Therefore, in this study, the potential of virgin and calcined basil seed on the adsorption of paraquat and diquat was investigated. The effects of contact time and initial pesticide concentration were also evaluated. In addition, simulated intestinal fluid was used to assess the application of basil seed in an *in vitro* system.

## 2 Materials and methods

### 2.1 Materials

Raw basil seed (BS) and medicinal carbon (MC) were obtained from Thai Cereal World Co., Ltd. (Thailand) and Nichi-Iko Pharm. Co., Ltd. (Japan), respectively. Calcined BS at 1000 °C (BS1000) was prepared by the carbonization of BS (Uematsu et al., 2020). Paraquat dichloride standard (C_12_H_14_C_l2_N_2_) and diquat dibromide monohydrate standard (C_12_H_12_Br_2_N_2_•H_2_O) were purchased from FUJIFILM Wako Pure Chemical Co., Japan. The commercial product (7% diquat dibromide +5% paraquat dichloride) was obtained from Syngenta Co., Ltd., Japan. Concentrations of paraquat and diquat were measured using previously reported methods (Nishida et al., 2005). Moreover, we confirmed that there was no change in the maximum absorption wavelength before and after the adsorption treatment in a preliminary experiment (the maximum absorption wavelengths of paraquat and diquat are 603 and 432 nm, respectively).

The physicochemical characteristics of the prepared tested samples were determined using the following methods. The morphology of the tested samples were determined using a scanning electron microscope (SU1510; Hitachi High-Technologies Co., Japan), with 5 μm of a beam diameter and 15 kV of voltage. The thermogravimetric-differential thermal analysis was conducted using a thermal gravimetric differential thermal analyzer (TG8120; Rigaku Co., Japan) under atmospheric air, with a temperature elevation rate of 10 °*C min*^−1^. The sample surface characteristics such as specific surface area and pore volume were measured using a specific surface area analyzer (NOVA4200*e*; Yuasa Ionics, Japan), based on nitrogen adsorption/desorption at liquid nitrogen temperature. In addition, the surface functional groups and the pH point of zero charge (pH_pzc_) were determined using the method reported by Boehm (1966) and Faria et al. (2004), respectively. Finally, to elucidate the interaction between the BS samples and pesticides, the binding energy or elemental distribution were analyzed using an X-ray photoelectron spectrometer (AXIS-NOVA; Shimadzu Co., Ltd., Japan), with 15 keV of a voltage and 10 mA of a current or a field emission electron probe microanalyzer (JXA-8530F; JEOL Ltd., Japan), with 15 keV of a voltage and 5 μm of a beam diameter, respectively.

### 2.2 Quantity of pesticides adsorbed using BS, BS1000, and MC

The removal of each pesticide using BS, BS1000, and MC was evaluated. Briefly, 0.05 g of each adsorbent was mixed with either 50 mg L^−1^ of paraquat or 70 mg L^−1^ of diquat solution in the volume of 50 mL, and then shaken at 100 rpm and 25 °C for 24 h. After adsorption, the tested samples were separated from a reaction mixture using a 0.45 μm membrane filter. The obtained filtrate was quantified and calculated for pesticides adsorbed, which comparable between before and after adsorption. In addition, the same experiment was repeated using a binary solution system (50 mg L^−1^ paraquat and 70 mg L^−1^ diquat).

### 2.3 Effect of contact time and initial herbicide concentration on the adsorption of pesticides

Initially, to evaluate the effect of contact time, 0.05 g of each tested adsorbent was mixed with either 50 mg L^−1^ of paraquat or 70 mg L^−1^ of diquat solution in the volume of 50 mL, and then shaken at 100 rpm and 25 °C for 0.5, 1, 1.5, 2, 3, 4, 5, 6, 20, and 24 h. Subsequently, to evaluate the effect of concentration, 0.05 g of each tested adsorbent and 50 mL of either paraquat or diquat solution were mixed at different initial concentrations, and then shaken at 100 rpm and 25 °C for 24 h. The quantity of each pesticide adsorbed was calculated using the method described in Section 2.2.

### 2.4 Application of BS and BS1000 for the removal of paraquat and diquat in simulated intestinal fluid

First, simulated intestinal fluid was prepared for the disintegration test using the method reported in The 17^th^ Japanese Pharmacopoeia (The Japanese Pharmacopoeia, 2016). This test was harmonized with those described in the European Pharmacopoeia and the U.S. Pharmacopoeia. Next, the removal of paraquat and diquat in simulated intestinal fluid was demonstrated. Briefly, 0.05 g of each tested adsorbent and 50 mL of 50 mg L^−1^ paraquat (or 70 mg L^−1^ diquat) in simulated intestinal fluid were mixed, and then shaken at 100 rpm and 25 °C for 24 h. The quantity of each pesticide adsorbed was calculated based on the method described in Section 2.2.

## 3 Results and discussion

### 3.1 Physicochemical characteristics of BS, BS1000, and MC

**Figure 1:**
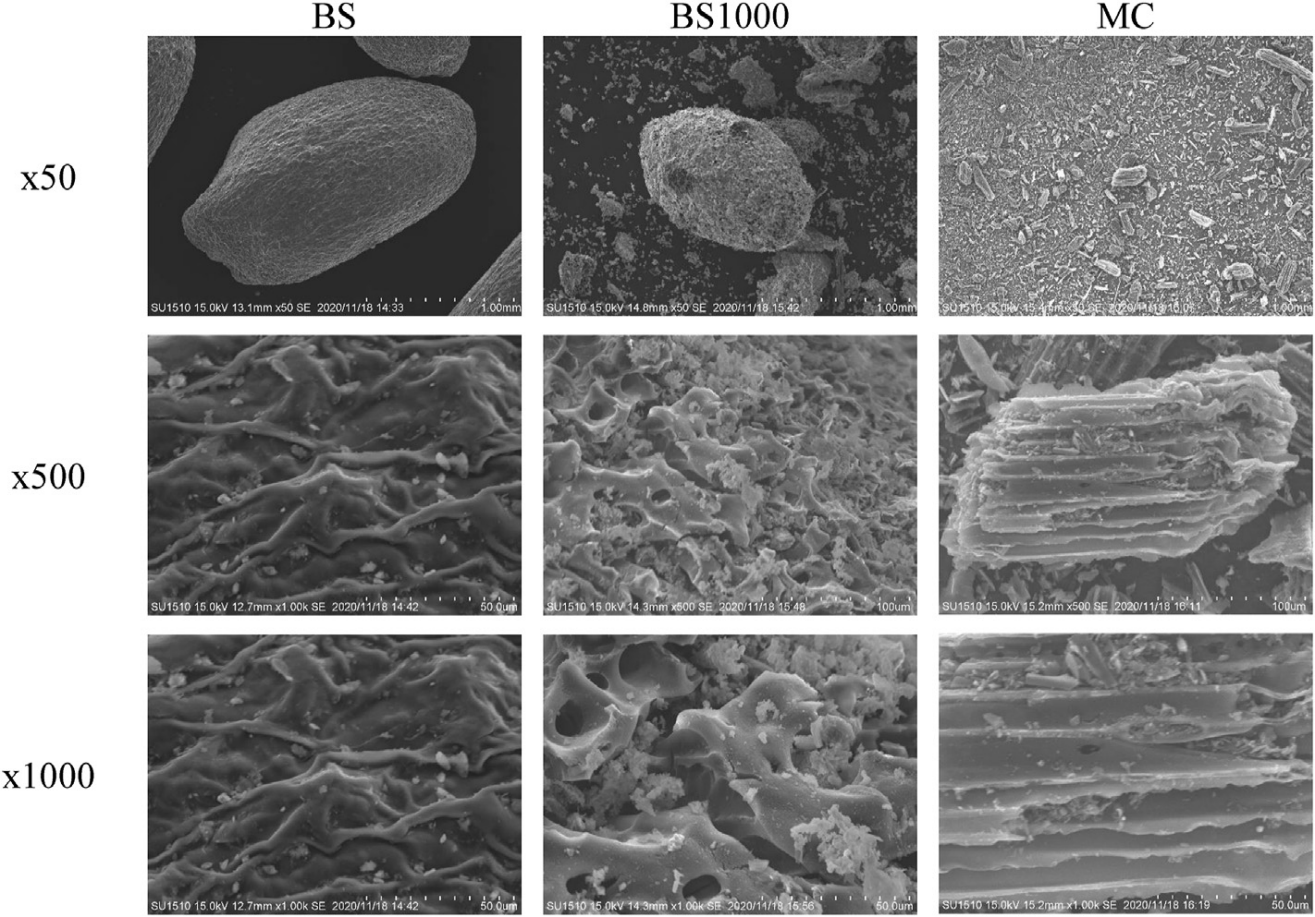
SEM images of each adsorbent.

**Figure 2:**
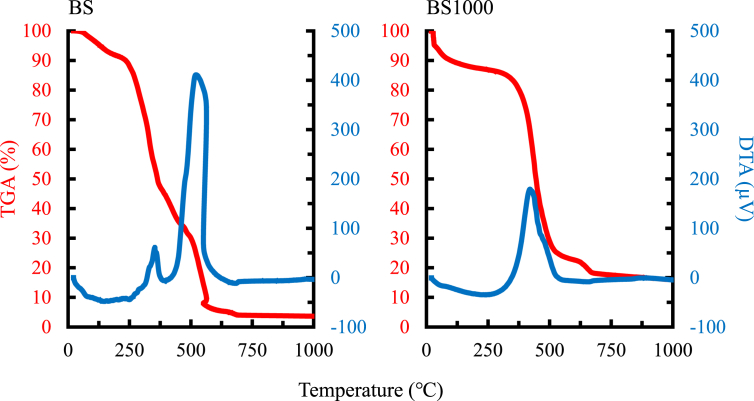
Thermal analysis of adsorbents under atmospheric air.

**Figure 3:**
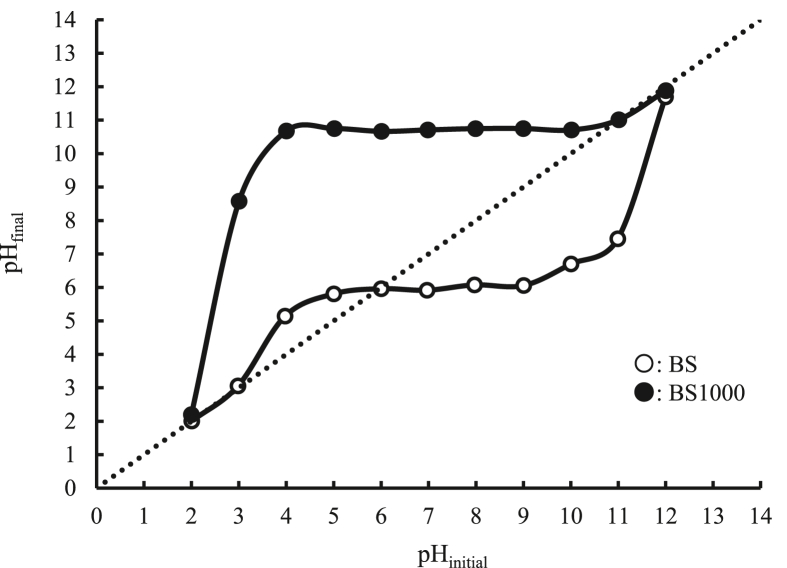
Determination of point of zero charge of BS and BS1000.

The physicochemical properties of raw and calcined basil seed have been reported previously (Uematsu et al., 2020). Here, the characteristics of the adsorbents are described briefly. The morphologies of BS, BS1000, and MC are illustrated in Figure 1. A structural collapse was observed in BS1000, and a number of pores were considerably increased with a step up in calcination temperature. Additionally, the morphology of the MC surface was quite different from that of BS and BS1000. The thermal analysis of BS and BS1000 is presented in Figure 2. Both a decrease in TGA and an increase in DTA occurred at the same time at a temperature range of 300–600 °C. Similar changes have been reported previously (Yokoyama et al., 2008), which indicate that the removal of surface functional groups such as carboxylic, phenolic, and other groups with the current treatment. Therefore, the surface characteristics of prepared adsorbents are presented in Table 1 and Figure 3. The results demonstrated that the 86.902 m^2^ g^−1^ on specific surface area of BS1000 was higher than that of BS (0.265 m^2^ g^−1^), and the concentration of surface functional groups of BS1000 (0.193 mmol g^−1^) was lower than that of BS (0.655 mmol g^−1^). These results are consistent with those mentioned earlier. In comparison, the specific surface area of MC was significantly greater than that of either BS or BS1000. However, the numbers of micropores were comparable to those of BS and BS1000 under the current conditions. The pH point of zero charge (pH_pzc_) is pH value at which the surface charge components become equal to zero under given conditions (Bakatula et al., 2018). This means that there are equal amounts of positive and negative charges on the adsorbent surface in this study. Therefore, the charge of the adsorbent surface is found to be clearly influenced by the solution pH, being positive at pH values lower than the pH_pzc_ and negative at pH values higher than the pH_pzc_. The pH_pzc_ values of BS, BS1000, and MC were 5.59, 11.01, and 6.26, respectively.

**Table 1:** Physicochemical properties of adsorbents.

Adsorbents	Specific surface area (m^2^ g^-1^)	Pore volume (cc g^-1^)			pH_pzc_	Surface functional groups (mmol g^-1^)	
		Micro	Meso	Macro		Acidic	Basic
BS	0.265	N.A.	N.A.	N.A.	5.59	0.050	0.605
BS1000	86.902	N.A.	0.019	0.005	11.01	0.006	0.187
MC	932.0	0.024	0.169	0.157	6.26	0.04	0.002

### 3.2 Paraquat and diquat adsorption capability of BS, BS1000, and MC

**Figure 4:**
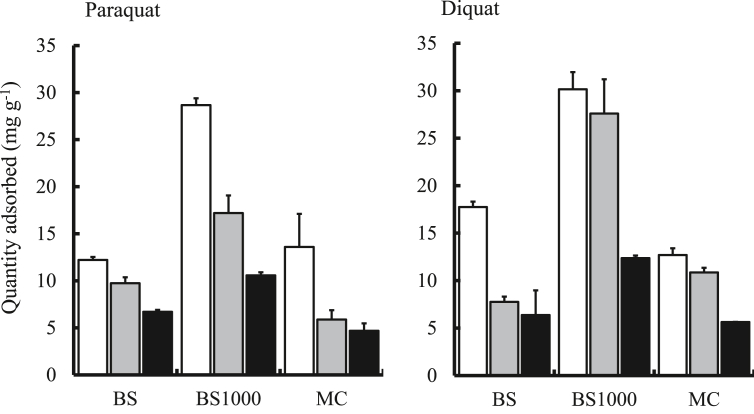
Quantity of paraquat and diquat adsorbed onto adsorbents. : Standard solution, : Commercial product, : Binary solution, initial concentration: 50 mg L^−1^ (paraquat) and 70 mg L^−1^ (diquat), sample volume: 50 mL, adsorbent: 0.05 g, temperature: 25 °C, contact time: 24 h, agitation speed: 100 rpm.

The quantity of paraquat and diquat adsorbed by BS, BS1000, and MC is shown in Figure 4. BS1000 adsorbed a greater quantity of paraquat and diquat than either BS or MC under the current conditions. These results suggest here that BS1000 prepared from waste biomass such as basil seed is a useful as bioadsorbent for the removal of herbicides from an aqueous medium. Moreover, the quantity of paraquat and diquat adsorbed in the binary solution system was smaller than that in the single solution, indicating that competition occurred easily between paraquat and diquat. Next, BS1000 showed the capability of adsorbing the commercial product of paraquat and diquat. However, the quantity of herbicide adsorbed with the commercial product was smaller than that with either the single or binary solution system. The presence of components in addition to paraquat and diquat (such as additives including minor components) affected the herbicide adsorption capability of BS1000. Therefore, further investigations are necessary to apply BS1000 for the removal of herbicides from the aqueous phase.

The comparison of paraquat and diquat adsorption capability of tested bio-adsorbent including BS, BS1000, and other reported adsorbents is shown in Table 2 (Duman et al., 2019; Kamga, 2019; Liang et al., 2021; Martins et al., 2015; Nakamura et al., 1993, 2000; Pavlovic et al., 2014). Regarding paraquat, the adsorption capability using BS1000 (28.7 mg g^−1^) was similar to or greater than that using other reported adsorbents (7.5–30 mg g^−1^). In addition, the adsorption capability of diquat using BS1000 was also greater than that using other reported adsorbents (except for OMWCNT and pristine g–CN–0.3). Therefore, BS1000 would be a potential candidate agent for the removal of herbicides such as paraquat and diquat from aqueous media.

**Table 2:** Comparison of quantity of paraquat or diquat adsorbed with previous researches.

Samples	Adsorbents	Quantity adsorbed (mg g^-1^)	Initial concentration (mg L^-1^)	pH	Temp. (°C)	Contact time (h)	Ref.
Paraquat	Spent coffee grounds	27.9	1000	7.0	-	1.0	Pavlovic et al. (2014)
	Kaolin clay	7.5	50	-	23	24	Martins et al. (2015)
	Chitosan beads	16.9	800	-	37	1.0	Nakamura et al. (1993)
	Wood sawdust	30	500	6.3–6.5	25	1.0	Kamga (2019)
	BS	13.0	50	-	25	24	This research
	BS1000	28.7	50	-	25	24	This research
Diquat	OMWCNT	58.3	16.3	6.5	25	24	Duman et al. (2019)
	OMWCNT-Fe_3_O_4_	20.9	16.3	6.5	25	24	Duman et al. (2019)
	OMWCNT-k-carrageenan-Fe_3_O_4_	10.7	16.3	6.5	25	24	Duman et al. (2019)
	Pristine g–CN–0.3	159.3	50	7	25	1.7	Liang et al. (2021)
	Activated carbon	Approximately 18	100	-	37	48	Nakamura et al. (2000)
	BS	17.8	50	-	25	24	This research
	BS1000	30.2	50	-	25	24	This research

**Figure 5:**
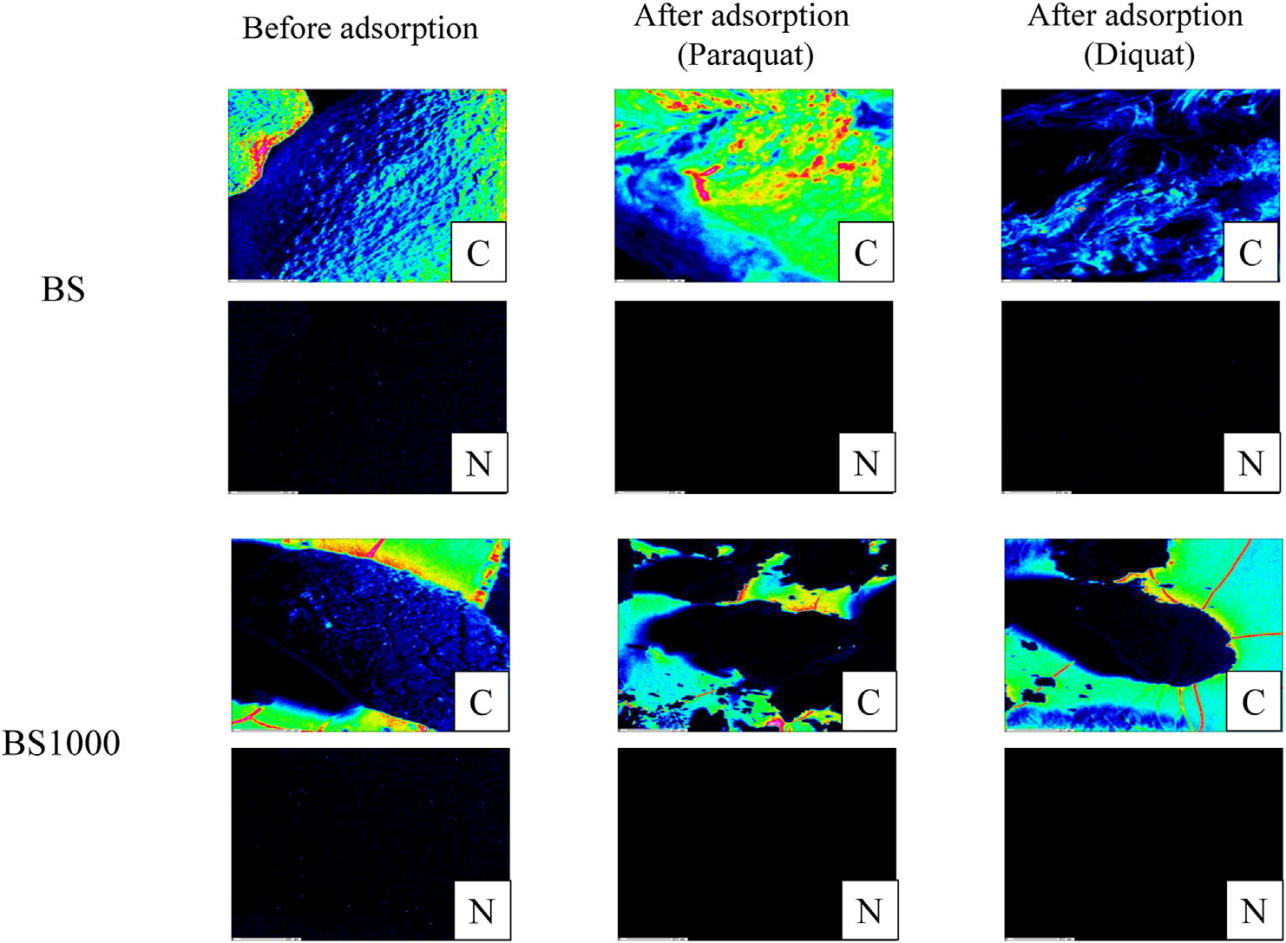
Elemental distribution of BS and BS1000 surface before and after adsorption. Low concentrationHigh concentration.

**Figure 6:**
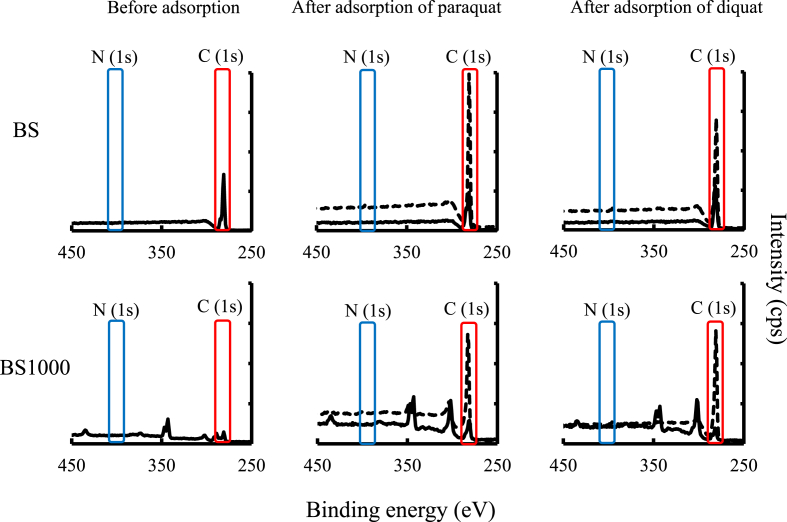
Binding energy of carbon and nitrogen onto adsorbent surface before and after adsorption. A solid line and a dotted line showed untreated and finely crushed adsorbent in after adsorption.

To elucidate the one of the adsorption mechanisms of paraquat and diquat using BS and BS1000, the elemental distribution and binding energy on the surface of each BS and BS1000 before and after adsorption were evaluated in this study as exhibited in Figures 5 and 6, respectively. The intensity concentration of C on the surface of adsorbent increased significantly after the adsorption of paraquat and diquat. However, the concentration of N did not change before and after adsorption owing to the relatively low content of N in paraquat and diquat. Therefore, the binding energies of C and N onto the surface of tested adsorbent before and after adsorption were assessed. Under the current conditions, the binding energies of C and N onto the untreated adsorbent surface were not changed after adsorption. However, the concentration of these elements on finely crushed adsorbent increased significantly after adsorption, which indicates that these herbicides are adsorbed by the adsorbent owing to characteristics such as pores.

### 3.3 Effect of contact time on the removal of paraquat and diquat using BS and BS1000

**Figure 7:**
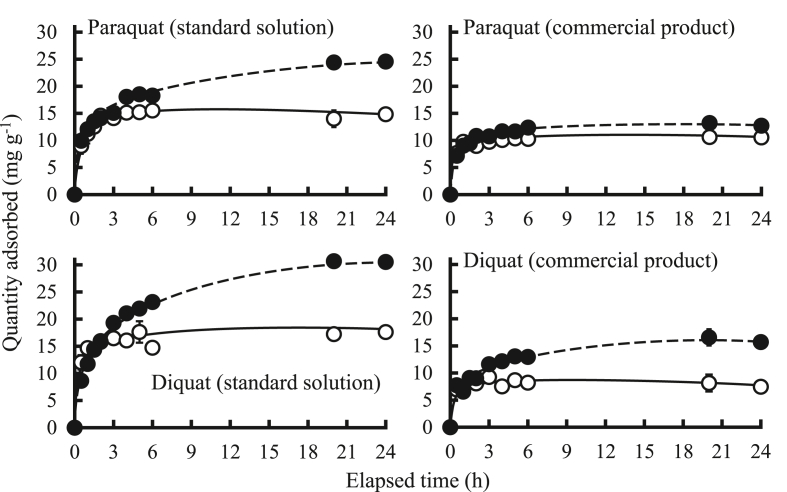
Effect of contact time on the adsorption of paraquat and diquat. : BS, : BS1000, initial concentration: 50 mg L^−1^ (paraquat) and 70 mg L^−1^ (diquat), sample volume: 50 mL, adsorbent: 0.05 g, temperature: 25 °C, contact time: 0.5, 1, 1.5, 2, 3, 4, 5, 6, 20, and 24 h, agitation speed: 100 rpm.

To elucidate the effect of contact time, the changes in adsorption capability of herbicides with contact time using BS and BS1000 were evaluated (Figure 7). It was observed that the quantity of adsorbed herbicides increased rapidly until 3 h from the start of adsorption, and then relatively slowed down. These changes are ascribed to the initial availability of a large number of free adsorption sites on the adsorbent surface and thereafter, the availability of fewer vacant sites may be responsible for the slow increment in the current conditions. Similar trends were reported previously (Kumari et al., 2019). Moreover, to obtain the information regarding the adsorption process relating to chemical reactions, mass transfer, and the adsorption order, pseudo-first-order and pseudo-second-order models were employed (Junthip et al., 2019).

The pseudo-first-order equation (Lagergren equation Eq. (1)) (Lagergren, 1898) applied in its linear form, and the pseudo-second-order equation (Eq. (2)) (Ho and McKay, 1999) can be expressed as:

(1) ln(*q*_*e*_−*q*_*t*_) = ln*q*_*e*_−*k*_1_*t*

(2) $$\frac{t}{q_{t}}=\frac{t}{q_{e}}+\frac{t}{k_{2}\times q_{e}^{2}}$$

where *q*_*e*_ (mg g^−1^) is the quantity of adsorbed herbicides at equilibrium, *q*_*t*_ (mg g^−1^) is the quantity adsorbed at time *t*, *k*_1_ (h^−1^) is the overall constant in the pseudo-first-order model, and *k*_2_ (g mg^−1^ h^−1^) is the pseudo-second-order adsorption constant.

**Figure 8:**
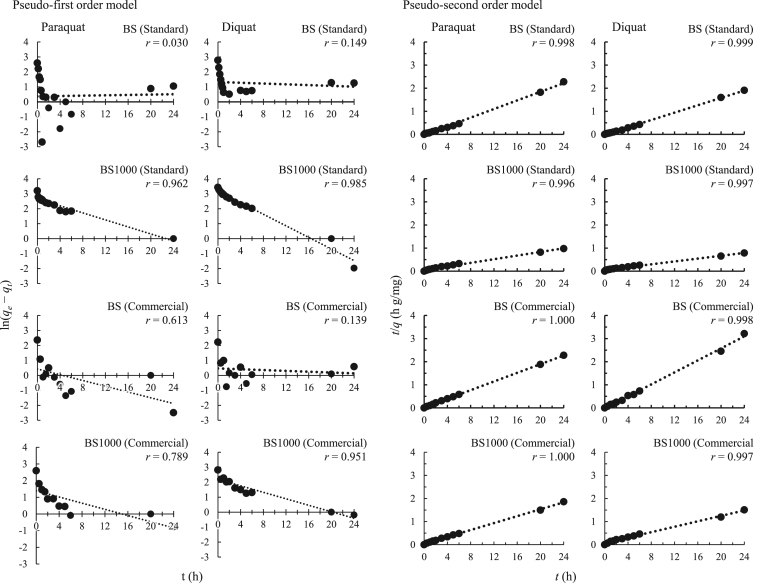
Pseudo-first- and pseudo-second-order plots for the adsorption of paraquat and diquat.

Table 3 and Figure 8 show the fitting results of kinetic data using the pseudo-first-order and pseudo-second-order models. The application of the pseudo-second-order equation showed excellent fitting to obtained results with the correlation coefficient more than 0.996 comparable to the pseudo-first-order model with the correlation coefficient ranged from 0.030–0.985. Additionally, the *q*_*e,exp*_ values of herbicides in the standard solutions and commercial product were closer to the *q*_*e,cal*_ value in the pseudo-second-order model than that of the pseudo-first-order model. These results demonstrate that the pseudo-second-order equation is better than the pseudo-first-order equation to explain the removal kinetics involved in the retention of paraquat and diquat ion-associates by BS and BS1000 with time (Vinhal et al., 2017).

**Table 3:** Fitting results of kinetic data using pseudo-first-order model and pseudo-second-order model.

Samples		Adsorbents	q_e,exp_ (mg g^-1^)	Pseudo-first-order model			Pseudo-second-order model		
				k_1_ (h^-1^)	q_e,cal_ (mg g^-1^)	r	k_2_ (g mg^-1^ h^-1^)	q_e,cal_ (mg g^-1^)	r
Standard solution	Paraquat	BS	13.4	-5.9 × 10^-3^	1.4	0.030	-3.9 × 10^-1^	10.7	0.998
		BS1000	24.6	1.2 × 10^-1^	14.3	0.962	3.5 × 10^-2^	25.3	0.996
	Diquat	BS	16.1	1.3 × 10^-2^	3.8	0.149	-4.4 × 10^-1^	12.5	0.999
		BS1000	30.7	1.9 × 10^-1^	24.0	0.985	1.9 × 10^-2^	32.5	0.997
Commercial product	Paraquat	BS	10.6	9.6 × 10^-2^	1.5	0.613	5.1 × 10^-1^	10.7	1.000
		BS1000	13.4	9.3 × 10^-2^	4.0	0.789	1.7 × 10^-1^	13.3	1.000
	Diquat	BS	9.3	1.4 × 10^-2^	1.6	0.139	-8.0 × 10^-1^	7.7	0.998
		BS1000	16.8	1.1 × 10^-1^	8.9	0.951	5.0 × 10^-2^	17.0	0.997

### 3.4 Effect of initial concentration on the removal of paraquat and diquat using BS and BS1000

**Figure 9:**
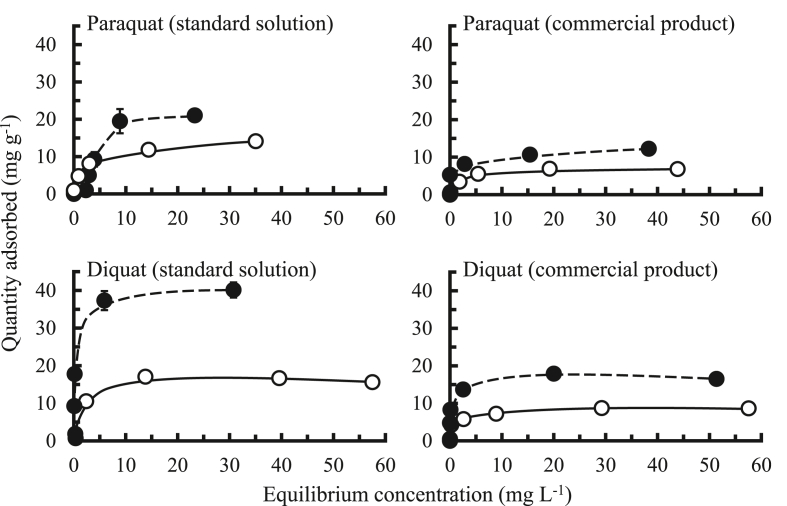
Adsorption isotherms of paraquat and diquat onto adsorbents. : BS, : BS1000, initial concentration: 0.5, 1, 5, 10, 25, and 50 mg L^−1^ (paraquat) and 0.7, 1.4, 7, 14, 35, and 70 mg L^−1^ (diquat), sample volume: 50 mL, adsorbent: 0.05 g, temperature: 25 °C, contact time: 24 h, agitation speed: 100 rpm.

Adsorption isotherms of paraquat and diquat using BS and BS1000 are shown in Figure 9. The quantity of herbicides adsorbed using BS1000 was greater than that using BS. In particular, the adsorption capability of herbicides from the commercial product using BS1000 was useful under the current conditions, which indicates that BS1000 is a potential agent for application in the field.

The adsorption isotherm models are represented through mathematical expression (Kumari et al., 2019). The relationship between herbicides and tested adsorbent in the aqueous phase can be described with the help of these two models. The Langmuir model is based on the homogeneity of the adsorbent. Differently, the Freundlich model is based on the heterogeneity of the adsorbent (Aouada et al., 2009). The Langmuir and Freundlich equations are described by Eqs. (3) and (4), respectively.

(3) $$1/q=1/\left(q_{\max}K_{L}C_{e}\right)+1/q_{\max}$$

(4) $$\log\;q=\frac{1}{n}\log C_{e}+\log K_{F}$$

where *q* is the quantity of adsorbed herbicides (mg g^−1^), *q*_*max*_ is the maximum quantity of herbicides adsorbed (mg g^−1^), and *C*_*e*_ is the equilibrium concentration (mg L^−1^). The removal capacity and strength of removal are *K*_*F*_ and 1/*n*, respectively. Additionally, *K*_*L*_ is the Langmuir isotherm constant (binding energy) (L mg^−1^).

**Figure 10:**
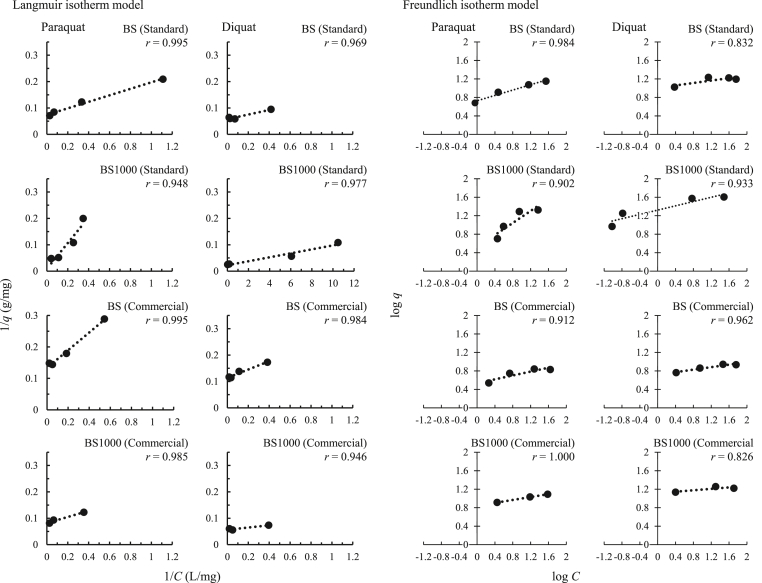
Langmuir and Freundlich isotherms plots for the adsorption of paraquat and diquat.

Table 4 and Figure 10 summarize the Langmuir and Freundlich constants for the adsorption of paraquat and diquat. Based on the fitting adsorption isotherm curves of these two models, with the use of BS1000, the correlation coefficient in the Langmuir isotherm model was ranged from 0.946–0.995 which was greater than that in the Freundlich isotherm model (0.826–0.984), except for paraquat in the commercial product. Therefore, monolayer adsorption for paraquat and diquat dominated on the homogeneous surface of BS and BS1000 (Junthip et al., 2019). Additionally, the *q*_*max*_ value in BS1000 was greater than that in BS for each herbicide. These trends were consistent with the adsorption isotherm data presented in Figure 9. Finally, when the 1/*n* value is 0.1–0.5, the adsorption of herbicides occurs easily; in contrast, when 1/*n* values are above 2, adsorption of herbicides is difficult. In this study, the 1/*n* value ranged from 0.1 to 0.7, indicating that the removal of paraquat and diquat using BS and BS1000 is useful (Abe et al., 1976).

**Table 4:** Langmuir and Freundlich constants for the adsorption of paraquat and diquat.

Samples		Adsorbents	Adsorption capacity (mg/g)	Langmuir isotherm model			Freundlich isotherm model		
				K_L_ (L mg^-1^)	q_max_ (mg g^-1)^	r	K_F_	1/n	r
Standard solution	Paraquat	BS	14.1	6.0 × 10^-1^	13.5	0.995	5.3	0.3	0.984
		BS1000	21.0	1.9 × 10^-2^	110.1	0.948	3.3	0.7	0.902
	Diquat	BS	16.7	6.6 × 10^-1^	17.3	0.969	10.2	0.1	0.832
		BS1000	40.0	3.0	44.3	0.977	21.0	0.2	0.933
Commercial product	Paraquat	BS	6.8	4.7 × 10^-1^	7.5	0.995	3.4	0.2	0.912
		BS1000	12.2	7.0 × 10^-1^	12.3	0.985	6.9	0.2	1.000
	Diquat	BS	8.7	7.2 × 10^-1^	8.8	0.984	5.2	0.1	0.962
		BS1000	16.5	1.3	17.7	0.946	13.0	0.1	0.826

### 3.5 Application of BS and BS1000 in simulated intestinal fluid

**Figure 11:**
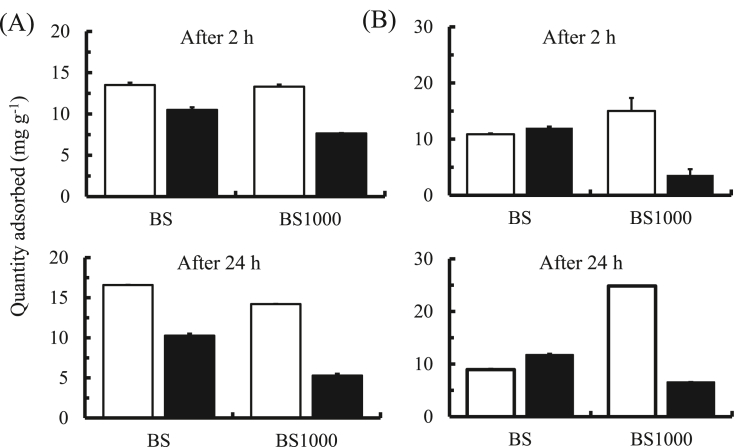
Quantity of paraquat (A) or diquat (B) adsorbed onto adsorbent in simulated intestinal fluid. : Standard solution, : Commercial product, initial concentration: 50 or 70 mg L^−1^, sample volume: 50 mL, adsorbent: 0.05 g, temperature: 25 °C, contact time: 2 and 24 h, agitation speed: 100 rpm.

To evaluate the application of BS and BS1000 for the removal of herbicides in the field, the adsorption capability of paraquat and diquat in simulated intestinal fluid was assessed (Figure 11). Considering paraquat adsorption, BS and BS1000 showed an excellent adsorption capability under the current conditions. The adsorption capacity of BS was greater than that of BS1000. In addition, the quantity adsorbed 2 h after the start of adsorption was not significantly different from that at 24 h.

Regarding diquat adsorption, BS and BS1000 both showed an adsorption capacity for the herbicides. However, the quantity of adsorbed diquat using BS1000 24 h after the start of adsorption was slightly higher than that 2 h after the start of adsorption under the experimental conditions. In summary, further studies are needed to elucidate in detail the adsorption capacity and adsorption mechanism of herbicides using BS and BS1000. However, these results provide fundamental information regarding the removal of herbicides such as paraquat and diquat from aqueous media using basil seeds as waste biomass.

## 4 Conclusion

BS and BS1000 were found to be candidates for the adsorption of herbicides from aqueous media and artificial intestinal juice. In addition, the quantity adsorbed using BS1000 was greater than that using BS. The binding energy of carbon (C1s) derived from herbicides was detected at approximately 280 eV after adsorption. These results aid in the elucidation of likely adsorption mechanisms, and the use of basil seed waste biomass as a potential resource for the removal of herbicides in an *in vitro* system.

## Declarations

### Author contribution statement

Yugo Uematsu: Performed the experiments; Analyzed and interpreted the data; Contributed reagents, materials, analysis tools or data; Wrote the paper.

Fumihiko Ogata: Conceived and designed the experiments; Analyzed and interpreted the data; Contributed reagents, materials, analysis tools or data; Wrote the paper.

Noriaki Nagai, Chalermpong Saenjum & Takehiro Nakmura: Performed the experiments; Contributed reagents, materials, analysis tools or data.

Naohito Kawasaki: Conceived and designed the experiments; Contributed reagents, materials, analysis tools or data.

### Funding statement

This research did not receive any specific grant from funding agencies in the public, commercial, or not-for-profit sectors.

### Data availability statement

Data included in article/supplementary material/referenced in article.

### Declaration of interests statement

The authors declare no conflict of interest.

### Additional information

No additional information is available for this paper.
